# Raman spectroscopy in halophile research

**DOI:** 10.3389/fmicb.2013.00380

**Published:** 2013-12-10

**Authors:** Jan Jehlička, Aharon Oren

**Affiliations:** ^1^Institute of Geochemistry, Mineralogy and Mineral Resources, Charles University in PraguePrague, Czech Republic; ^2^Department of Plant and Environmental Sciences, The Institute of Life Sciences, The Hebrew University of JerusalemJerusalem, Israel

**Keywords:** Raman spectroscopy, halophilic, salterns, gypsum crusts, compatible solutes, carotenoids

## Abstract

Raman spectroscopy plays a major role in robust detection of biomolecules and mineral signatures in halophile research. An overview of Raman spectroscopic investigations in halophile research of the last decade is given here to show advantages of the approach, progress made as well as limits of the technique. Raman spectroscopy is an excellent tool to monitor and identify microbial pigments and other biomolecules in extant and extinct halophile biomass. Studies of bottom gypsum crusts from salterns, native evaporitic sediments, halite inclusions, and endoliths as well as cultures of halophilic microorganisms permitted to understand the content, distribution, and behavior of important molecular species. The first papers describing Raman spectroscopic detection of microbiological and geochemical key markers using portable instruments are highlighted as well.

## Introduction

Laser Raman spectroscopy determines the wavelength and strength of inelastically dispersed light from molecules and their arrangement. Much of the laser light is scattered without change in wavelength (Rayleigh scattering), but part of light is dispersed differently and scattered light wavelength is shifted (Raman scattering). Measurements of these wavelength shifts create a unique Raman spectrum for each substance, providing information on chemical bonds and molecular and crystalline structure. Raman spectroscopy is therefore a rapid method of identifying organic compounds, it is generally considered to be non-destructive, and sample preparation is minimal (Schrader et al., 1999; Rösch et al., 2003; Schmitt and Popp, 2006; Jehlička and Edwards, 2008).

Many halophiles are pigmented by carotenoids or carotenoid-like pigments, and these have particularly strong Raman resonance signals (Merlin, 1985). Raman spectroscopy studies of pigmented halophiles in culture, have included red archaea of the family *Halobacteriaceae*, the β-carotene-rich alga *Dunaliella salina*, and *Salinibacter ruber*, a member of the phylum *Bacteroidetes*. The information gained was used to characterize natural communities of halophilic microorganisms in saltern brines and of halophiles trapped in fluid inclusions within halite crystals, recent as well as ancient. The high spatial resolution (~1 μm) at which data can be collected makes the method particularly useful in such studies. As halite does not give Raman signals, its presence does not interfere. Raman spectroscopy was also used to characterize the microbial communities colonizing evaporitic crusts such as gypsum precipitating in saltern evaporation ponds and halite in desert environments.

Another application for Raman spectroscopy is the assessment of the presence of organic osmotic solutes within halophilic microorganisms. Such organic, “compatible” solutes are expected to be present in high concentrations within the cells to provide osmotic equilibrium with the outside salt concentration (Roberts, 2000; Oren, 2013b). Therefore, Raman signals attributed to such compounds are expected to be relatively easily detectable, even if typical osmotic solutes such as glycerol, glycine betaine, ectoine, etc. do not allow observing resonance Raman effects at the same sensitivity as carotenoid pigments.

Such studies are also highly relevant for astrobiology. Miniaturized Raman spectroscopes are planned to be included in a Mars lander in the near future. Hypersaline and/or hyperarid environments on Earth are excellent model systems to explore the possibilities and limitations of Raman spectroscopy for the detection of life in general, and more specifically, for the detection of halophilic microorganisms, modern or ancient that may possess carotenoids or other protective pigments, and/or high concentrations of organic osmotic solutes (Vítek et al., 2012; Winters et al., 2013).

## Raman analysis of microbial pigments in evaporites

The saltern ponds near Eilat (Red Sea coast of Israel) were used as a model system for several Raman spectroscopy studies. The upper layer (down to a few cm) of the gypsum crust that accumulates on the bottom of evaporation ponds of intermediate salinity (150–200 g/l total salts) is yellow-orange, due to the presence of unicellular cyanobacteria (*Aphanothece*-type), below this a green layer with filamentous *Phormidium*-type cyanobacteria is found, and a third layer colored red-purple by purple sulfur bacteria (*Halochromatium*-type) (Oren et al., 1995; 2009). Pigments expected to be present include carotenoids such as echinenone and myxoxanthophyll in the cyanobacterial layers, chlorophyll *a* and phycocyanin (mainly in the green layers), and bacteriochlorophyll *a* and spirilloxanthin carotenoids in the purple layer (Oren et al., 1995; Oren, 2011). We have used a portable Raman spectrometer with 532 nm excitation laser for fast screening of these pigments in the field. The light handheld instrument allowed obtaining acceptable quality spectra fast and outdoors. Raman bands at 1513, 1153, 1005 cm^−1^ recorded in the orange layer are assumed to be related to echinenone. Chlorophyll *a* and phycocyanin expected in the green layer were not detected with the green excitation. Raman spectra of the red layer contain major bands at 1510, 1151, and 1004 cm^−1^, documenting the presence of spirilloxanthin-like carotenoids (Jehlička and Oren, 2013).

Portable Raman spectrometers were also used to obtain information on microbial pigments within dry gypsum and halite evaporites in the hyperarid regions of the Atacama Desert, Chile. The halite samples were colonized by halophilic and halotolerant cyanobacteria; gypsum crusts contained cyanobacteria as well as *Trebouxia*-like algal cells. Measurements were performed directly on the rock as well as on the homogenized, powdered samples. Excitation at 532 nm was found superior for the analysis of powdered specimens due to its high sensitivity toward. However, the 785 nm excitation wavelength enabled more sensitive detection of the UV-protecting pigment scytonemin found in the sheath of many desert cyanobacteria (Edwards et al., 2005; Wierzchos et al., 2006; de los Ríos et al., 2010; Jehlička et al., 2011; Vítek et al., 2012, 2013). Raman spectroscopy combined with microscopic imaging of the phototrophic community in Atacama Desert gypsum crusts with cyanobacterial and algal colonization enabled the identification of β-carotene, other carotenoids and phycobiliproteins, and degradation of phycobiliproteins could be monitored (Vítek et al., 2013).

## Raman analysis of pigmented microorganisms in saltern crystallizer brines

NaCl-saturated brines of saltern crystallizers are typically inhabited by three types of caroteinoid-rich microorganisms: the green alga *Dunaliella*, archaea of the family *Halobacteriaceae* (e.g., *Haloquadratum walsbyi*), and the bacterium *Salinibacter ruber* (Oren and Dubinsky, 1994; Antón et al., 2002; Oren, 2009, 2013a,c). Similar red brines are prominent in the northern arm of Great Salt Lake (Utah, USA) and in other hypersaline lakes (Post, 1977; Oren, 1988).

Although Raman spectra of carotenoids have many features in common, there are also differences that can be used to discriminate between compounds. The C=C band is an indicator of conjugated chain length, which can therefore be used for diagnostic purposes: the wavenumber of the C=C mode is decreased in value as the conjugation length increases. Bacterioruberin of haloarchaea has a rather different molecular structure with a primary conjugated isoprenoid chain length of 13 C=C units with no subsidiary conjugation arising from terminal groups, which contain four OH group functionalities only. A first Raman study haloarchaeal carotenoids was published by Marshall et al. (2007), who suggested that another haloarchaeal pigment, bacteriorhodopsin, may be of interest as a potential biomarker for exobiology purposes. The Raman spectrum of pure bacteriorhodopsin shows a C=C bonds-related band at 1536 cm^−1^. We recently have provided a comparison of different members of the *Halobacteriaceae* (*Halobacterium salinarum*, *Halorubrum sodomense*, *Haloarcula vallismortis*) (Jehlička et al., 2013), and *Salinibacter ruber*, which harbors salinixanthin, a C_40_-carotenoid acyl glycoside (Lutnæs et al., 2002). We did not find any Raman signatures of bacteriorhodopsin in our preparations. A similar observation was reported by Fendrihan et al. (2009). Probably the bacteriorhopsin content in the samples examined was too low for detection. In the salinixanthin spectrum we found signals corresponding to a carotenoid with 11 C=C in the primary isoprenoid chain conjugation plus one more in a terminal alicyclic ring; the three strongest bands in the spectrum arise from the isoprenoid chain. Other assignments of salinixanthin resonance bands included evidence for the glycosidic stretching mode of the associated sugar ring at 1187 cm^−1^ and a COH stretching mode at 1041 cm^−1^ (Jehlička et al., 2013).

Based on the information obtained with pure cultures, it now became possible to analyze biomass collected from saltern crystallizer brines by Raman spectroscopy. Planktonic biomass was collected by centrifugation, a treatment that yields pellets of prokaryotic cells (*Halobacteriaceae*, *Salinibacter*), but removes the major part of β-carotene-containing *Dunaliella* cells as these do not sink during centrifugation (Oren and Dubinsky, 1994; Oren, 2009). Bacterioruberin was the major carotenoid in a cell pellet collected. No distinctive signals for salinixanthin were detected. This was expected: salinixanthin is always a minor component of the community, and in Eilat it was found in much smaller amounts than e.g., in similar saltern ponds in Spain (Oren and Rodríguez-Valera, 2001).

Raman spectroscopy thus has a great potential in assessing presence of different carotenoid compounds of microbial communities living at the highest salt concentrations (Jehlička et al., 2013). However, due to the large extent of chemical similarities and the potential overlap of Raman bands, the technique must be complemented with other techniques of separation and identification of carotenoids such as HPLC.

## Use of raman spectroscopy to characterize biomarkers and microorganisms trapped within recent and ancient salt crystals

When hypersaline brines are evaporated to yield halite, small amounts of brine are often included within the crystals. Halophilic microorganisms contained in the brine may become entrapped within the inclusions and survive there for prolonged periods (Schubert et al., 2009). There have been claims that microorganisms may be able to survive there for many millions of years.

As organisms inhabiting saturated brines are generally rich in carotenoid pigments, and as Raman microscopes have a high resolution to the scale of a few micrometers or less, and as halite does not interfere with the analysis, Raman spectroscopy is an excellent technique to monitor presence of microorganisms entrapped within salt crystals, modern as well as ancient. This idea was pioneered by Fendrihan et al. (2009), who compared Raman spectra of pure β-carotene to carotenoids within halophilic archaea (*Halococcus dombrowskii, Hcc. morrhuae, Halobacterium salinarum, Hbt. noricense, Haloarcula japonica, Halorubrum saccharovorum, Halorubrum chaoviator*) sealed in laboratory-grown halite crystals. The cells were embedded in halite by drying cell suspensions overnight on a glass microscope slide or on a quartz dish to simulate evaporitic conditions, and the crystals were investigated by FT-Raman spectroscopy using excitation at 1064 nm and dispersive microRaman spectroscopy at 514.5 nm. The spectra showed prominent peaks at 1507, 1152, and 1002 cm^−1^, attributed to bacterioruberins. Similar studies permitted to show how Raman microspectrometry can be used for detecting amino acids (glycine, L-alanine, β-alanine, L-serine) in water solutions contained in synthetic inclusions in halite (Osterrothová and Jehlička, 2011).

As a preparation for future deployment of Raman instruments for the search for traces of life on Mars (see section Implications for the Search for Halophilic Life on Mars), Raman microspectrometry was tested as a non-destructive method of determining the lowest detectable β-carotene content within evaporitic matrices: gypsum, halite, and epsomite. Signals were obtained at β-carotene concentrations down to the 0.1–10 mg kg^−1^ level in halite, the number Raman bands observed depending on the mineral matrix and the excitation wavelength used (514.5 nm being more sensitive than 785 nm for detection of carotenoids). Within gypsum and epsomite crystals, one order of magnitude higher concentrations were required for detection (Vítek et al., 2009).

Portable and miniaturized Raman spectrometers were also applied to the detection of microbial biomarkers in natural halite from the hyperarid region of the Atacama Desert. Measurements were performed directly on the rock and on homogenized, powdered rock samples. For carotenoid analysis, excitation at 532 nm excitation was found to be superior for the analysis of powdered specimens, but excitation at 785 nm enabled better detection of scytonemin, another pigment present in the material examined (Vítek et al., 2012).

A highly exciting application of Raman spectroscopy to detect life forms within salt crystals is the finding of carotenoids in brine inclusions in ancient halite, 9 ka to 1.44 Ma in age, from borehole cores from Death Valley, Saline Valley, and Searles Lake, CA. These carotenoids occurred within fluid inclusions as colorless to red-brown amorphous and crystalline masses associated with spheroidal algal cells resembling *Dunaliella*. Raman spectra of authentic carotenoid standards (β-carotene, lycopene, lutein) showed the same characteristic bands (1000–1020 cm^−1^, 1150–1170 cm^−1^, and 1500–1550 cm^−1^) as found in the ancient halite. Carotenoids appear well-preserved in ancient salt, a finding that supports reports of the recovery of preserved DNA and even of live cells from fluid inclusions in buried halite deposits (Winters et al., 2013).

## Raman spectra of organic osmotic solutes in bacterial cultures and in microbial communities in evaporitic crusts

A few halophiles (the haloarchaea, *Salinibacter*) use KCl to provide osmotic balance of their cytoplasm, but most other halophilic and halotolerant prokaryotic and eukaryotic microbes accumulate high concentrations of organic osmotic solutes (“compatible solutes”) to protect the cells against extreme osmotic pressures. This strategy of osmotic adaptation does not require extensive changes in the structure of intracellular enzymes. A great diversity of organic osmotic solutes has been identified in the microbial world. Examples are glycerol produced by *Dunaliella*, simple sugars (sucrose, trehalose), amino acids (proline, glutamic acid, β-glutamine), and amino acid derivatives [e.g., glycine betaine, ectoine ((S)-2-methyl-1,4,5,6-tetrahydropyrimidine-4-carboxylic acid)], 5-hydroxyectoine, and Nε-acetyl-β-lysine (Galinski et al., 1985; Wohlfarth et al., 1990; Severin et al., 1992; Galinski, 1995; Welsh, 2000; Roberts, 2006). There are solutes found thus far in one organism only, such as *N*-α-carbamoyl-L-glutamine 1-amide from *Ectothiorhodospira marismortui* (Galinski and Oren, 1991). Many microorganisms can accumulate more than one compatible solute. Glycine betaine is widely found in photosynthetic prokaryotes, oxygenic as well as anoxygenic, but few heterotrophic prokaryotes are able of its *de novo* biosynthesis. However, when available (e.g., in media containing yeast extract), many heterotrophs accumulate glycine betaine from the growth medium (Imhoff and Rodriguez-Valera, 1984; Oren, 2013b). Many moderately halophilic/halotolerant cyanobacteria produce the heteroside glucosylglycerol [2-*O*-α-D-glucopyranosyl-(1→2)-glycerol], while the most salt tolerant strains accumulate glycine betaine (Mackay et al., 1984; Hagemann, 2011).

As osmotic solutes must be present in high concentrations intracellularly to provide the necessary osmotic equilibrium, we hypothesized that they may form suitable biomarkers to be identified by Raman spectroscopy. We therefore prepared a database of Raman spectra of commonly encountered solutes, including ectoine, hydroxyectoine, glycine betaine, glucosylglycerol, mannosylglycerate (potassium salt), and di-myo-inositol phosphate, complementing existing information on the Raman spectra of solutes such as glycerol, sucrose, and trehalose (Jehlička et al., 2012a) (Table 1). The purpose was to establish a tool for the rapid assessment of the osmotic adaptation mechanism used by halophiles in culture and in natural settings, including the use of miniaturized portable Raman spectrometers to probe halophilic microbial communities in the field.

**Table 1:** **Main Raman bands and intensities for pigments and compatible solutes**.

**Compound**	**Origin**	**Prominent Raman bands observed**	**References**
**PIGMENTS**			
Bacterioruberin	*Halobacterium salinarum* NRC-1 culture	1505 s, 1152 s, 1000 s	Marshall et al., 2007
	*Halococcus dombrowskii* culture	1507 s, 1152 s, 1002 s	Fendrihan et al., 2009
	*Halorubrum sodomense* culture	1506 s, 1152 s, 1001 s	Jehlička et al., 2013
	*Haloarcula vallismortis* culture	1506 s, 1151 s, 1000 s	Jehlička et al., 2013
Salinixanthin	*Salinibacter ruber* culture	1512 s, 1155 s, 1003 s	Jehlička et al., 2013
Bacteriorhodopsin	Standard	1526 s, 1199 m, 1168 m, 1150 m, 1007 s	Marshall et al., 2007
Chlorophyll	Native crusts Atacama cyanobacteria	1445 m, 1308 m, 745 m, 508 m	Vítek et al., 2013
Scytonemin	*Lyngbya aestuarii* from native mat	1595 m, 1554 m, 1173 m	Edwards et al., 2000
	Native crusts Atacama	1596 s, 1558 s, 1321 s, 1172 m	Vítek et al., 2010
Phycobiliproteins	Native crusts Atacama	1631 w, 1583 w, 1370 w, 1283 w, 1235 w, 874 w, 815 w, 665 w	Vítek et al., 2013
**COMPATIBLE SOLUTES**			
Glycerol	Standard	1465 m, 1315 m, 1257 m, 1110 s, 1055 s, 923 m, 850 s, 820 s, 675 m, 485 m, 416 m, 392 s	De Gelder et al., 2007
Trehalose	Standard	1455 m, 1411 m, 1386 m, 1371 m, 1358 vs, 1330 m, 1149 vs, 1120 vs, 1102 s, 1080 m, 1061 m, 1018 m, 912 vs838 vs, 804 m, 697 m, 671 m, 604 m, 540 s, 523 vs, 450 s, 430 m, 407 vs, 369 vs	De Gelder et al., 2007
Glutamate	Standard	1434 m, 1421 s, 1401 vs, 1353 m, 1341 s, 1317 s, 1293 m, 1282 m, 1142 m, 1095 m, 942 vs, 925 m, 876 s, 857 vs, 775 m, 529 m, 499 m	De Gelder et al., 2007
L-proline	Standard	1454 s, 1389 m, 1378 m, 1286 m, 1267 m, 1240 m, 1083 m, 1045 m, 1035 m, 994 m, 947 m, 930 m, 916 vs, 899 vs, 877 m, 850 s, 842 vs, 834 vs, 642 m, 452 vs	De Gelder et al., 2007
Ectoine	Standard	3009 m, 2981 m, 2944 s, 2895 m, 1469 m, 1444 m, 1392 m, 1366 w, 1316 ms, 1280 ms, 1215 w, 1207 w, 1137 m, 1072 w, 1032 w, 999 w, 850 s, 804 vw, 692 vw, br, 657 vw, 599 w, 570 w, 498 w, 432 w	Jehlička et al., 2012a
	*Halomonas elongata* culture (minimal medium)	2985 m, sh, 2937 s, br, 2888 m, br, 1633 w, 1465 vw, sh, 1439 w, sh, 1394 s, 1319 vw, sh, 1275 m, 1212 w, sh, 1136 w, 1030 vw, 1000 vw, 903 vw, 845 m, 803 vw, 686 vw, 655 vw, 596 w, 571 w, 435 vw	Jehlička et al., 2012b
Hydroxyectoine	Standard	3017 m, 2935 s, 861 w, 1640 w, 1613 m, 1438 m, 1349 m, 1318 ms, 1263 s, 1213 m, 1151 m, 1092 ms, 1014 s, 970 w, 901 m, 890 m, 828 ms, 751 s, 631 m, 558 m, 395 m, 381 s, 359 m, 311 m	Jehlička et al., 2012a
Glycine betaine	Standard	3375 m, 3290 m, 3053 m, 3034 m, 2982 m, 2969 s, 2867 w, 2937 m, 1650 w, 1462 s, 1427 m, 1412 w, 1343 m, 1132 w, 953 w, 939 w, 895 m, 783 s, 539 m, 363 m	Ilczyszyn and Ratajczak, 1996
	*Halomonas elongata* culture (complex medium)	3050 w, 3031 m, 2978 m, 2965 s, 2942 w, 2935 m, 2885 w, 2880 w, 2864 w, 2784 m, 1613 vw, 1458 m, 1423 w, 1410 w, 1340 w, 1129 w, 974 m, 952 m, 933 s, 896 w, 780 s, 725 w, 538 m, 457 w, 361 m	Jehlička et al., 2012b
	*Ectothiorhodospira marismortui* culture	3059 w, 3032 m, 2966 m, 2936 m, 1645 vw, 1625 vw, 1457 m, 1410 vw, 1394 vw, 1342 vw, 1211 vw, 1127 vw, br, 976 w, 954 w, 933 s, 897 w, 779 s, 540 m, 456 w, 422 w, 366 w, 338 w	Jehlička et al., 2012b
	Native gypsum crust (Eilat, Israel, red layer)	3058 w, br, 3033 m, 2969 s, 2939 ms, 2880 m, 1458 m, 1452 m, 1421 w, sh, 1337 mW, 1212 w, 1131 vw, 978 w, 956 m, 934 s, 911 mW, 899 mW, 780 s, 730 vw, br, 684 vw, 542 mW, 458 w, 369 w, 339 vw	Oren et al., 2013
Glucosylglycerol		2930 br, 2907 s, 1460 s, 1366 s, br, 1340 s, br, 1265 w, br, 1127 m, 1103 w, 955 w, 897 m, 842 s, 755 w, 707 w, 666 w, 544 m, 514 m, 435 w	Jehlička et al., 2012a
Mannosylglycerate (K salt)		3585 m, 3405 m, 2975 m, 2956 m, 2938 s, 2931 s, 2906 m, 1461 m, 1353 m, 1308 m, 1153 m, 1135 m, 1107 m, 1093 m, 1069 s, 1055 m, 1004 m, 958 m, 892 s, 825 s, 671 m, 589 m, 517 s, 429 w, 390 m, 350 m	Jehlička et al., 2012a

s, strong bands; m, medium bands; vw, very weak bands; mw, weak bands; w, weak bands; br, broad bands; sh, shoulder.

We then tested for the presence of organic compatible solutes within cells of two model organisms, both members of the *Gammaproteobacteria*: the heterotrophic *Halomonas elongata* and the anoxygenic phototroph *Ectothiorhodospira marismortui*. We did not succeed detecting organic osmotic solutes directly in cell pellets, and therefore extraction and purification methods were found essential. Perchlorate extraction, followed by desalting on an ion retardation column and lyophilization, a method also recommended for analysis of compatible solutes by HPLC and NMR techniques (Wohlfarth et al., 1990; Severin et al., 1992; Roberts, 2006) yielded good results (Jehlička et al., 2012b). Because of the time and effort needed for sample preparation some of the normal advantages of Raman spectroscopy over other methods do not apply here. However, the rapid analysis by Raman spectroscopy, typically a few minutes only, remains a clear advantage. Near-infrared 785 nm lasers were found especially suitable for the identification of compatible solutes.

*H. elongata* is known to accumulate glycine betaine when grown in complex media containing yeast extract, but to produce ectoine in defined media on e.g., glucose as carbon and energy source (Imhoff and Rodriguez-Valera, 1984; Wohlfarth et al., 1990; Severin et al., 1992). Raman signals attributable to glycine betaine were found in extracts of cells grown in rich media. Extracts of *H. elongata* cells grown on glucose, in contrast, showed characteristic medium- and strong intensity bands permitting the unambiguous identification of the compatible solute present as ectoine (Jehlička et al., 2012b).

In desalted perchlorate extracts of *E. marismortui* a complete series of sharp bands corresponding to glycine betaine was recorded, including bands in the second order area. Trehalose was not found, nor were any signals obtained that may have corresponded to the minor osmotic solute *N*-α-carbamoyl-L-glutamine 1-amide (a compound not included yet in the library of Raman resonance signals) (Jehlička et al., 2012b).

The methodology developed for extraction and Raman analysis of compatible solutes in bacterial cultures (*Halomonas*, *Ectothiorhodospira*) (Jehlička et al., 2012b) was also applied to the analysis of the stratified microbial communities in an evaporitic gypsum crust found in an evaporation pond (~194 g/l total dissolved salts) of the salterns of Salt of the Earth Eilat (Oren et al., 1995; see also section Raman Analysis of Microbial Pigments in Evaporites). Solutes were extracted from the upper yellow-orange layer dominated by unicellular cyanobacteria, the green layer with filamentous cyanobacteria, and the layer colored red-purple by purple sulfur bacteria, all accompanied by dense communities of heterotrophic bacteria. Extracts were analyzed by Raman spectroscopy, by ^1^H and ^13^C nuclear magnetic resonance and by HPLC. The only osmotic solute detected in all three layers was glycine betaine; ectoine and other known solutes were not found. Most heterotrophic bacteria cannot produce glycine betaine but preferentially accumulate it when it is supplied. Presence of glycine betaine produced by the photoautotrophic members of the community may therefore relieve the heterotrophs from the need to synthesize other compounds at a high energetic cost (Oren et al., 2013).

## Implications for the search for halophilic life on mars

A miniaturized Raman spectrometer is scheduled to fly as part of the analytical instrumentation on an ESA remote robotic lander in the ESA/Roscosmos ExoMars mission, planned to be launched in 2018. Salts are present on Mars, including ancient salts formed in periods when the climate may have been more suitable for life than it is today. In view of the longevity of halophiles on Earth when entrapped within salt crystals, and the presence of carotenoid pigments detectable by Raman spectroscopy in such ancient salt samples (Fendrihan et al., 2009; Winters et al., 2013), Raman techniques may be excellent tools for life detection systems to be sent to Mars (Edwards et al., 2005, 2013; Vítek et al., 2009, 2012). Whether halophilic microorganisms resembling the current ones on Earth are or were present in the past on Mars is yet unknown, but if they are, Raman spectroscopy may well help us discovering them.

### Conflict of interest statement

The authors declare that the research was conducted in the absence of any commercial or financial relationships that could be construed as a potential conflict of interest.
